# Supplementary material for: Assessing anti-rabies vaccine response in humans: A rapid and high-throughput adaptable, pseudovirus-based neutralization assay as an alternative to rapid fluorescent focus inhibition test (RFFIT)
Source: PLoS Negl Trop Dis. 2025 Apr 10;19(4):e0013010. doi: 10.1371/journal.pntd.0013010 (PMC12013880; doi:10.1371/journal.pntd.0013010)
Supplement: S1 Table — (PDF) [file pntd.0013010.s001.pdf]

Assessing anti-rabies vaccine response in humans: A rapid and high-throughput-adaptable neutralization assay as an alternative to Rabies Fluorescence Focus Inhibition test (RFFIT)

Santhik S Lupitha[1,2], Geetu Rose Varghese[1], Lekshmi J Das[1], Priya Prabhakaran[2], Ashwini M. Ananda[3], Reeta S Mani[3]*, Easwaran Sreekumar[1,4]*

[1]Molecular Bioassay Laboratory; [2]Department of Virus Applications; Institute of Advanced Virology (IAV), Thonnakkal, Thiruvananthapuram 695317, Kerala, India

[3]Department of Neurovirology, WHO Collaborating Centre for Reference and Research in Rabies, National Institute of Mental Health and Neurosciences (NIMHANS), Bangalore; India

[4]Molecular Virology Laboratory, Rajiv Gandhi Centre for Biotechnology (RGCB), Thiruvananthapuram 695014, Kerala, India

Supplementary Table1: RFFIT and PVNT titres of human serum samples

Sample ID	RFFIT (IU/ml)	PVNT (IU/ml)
1	0	0
2	0	0
3	0	0
4	0	0
5	0	0
6	1.87	1
7	1.87	1.87
8	1.87	0.5
9	1.87	0.5
10	7.5	7.5
11	7.5	7.5
12	7.5	7.5
13	7.5	7.5
14	7.5	0.9

15	15	15
16	15	7.5
17	15	7.5
18	15	7.5
19	15	15
20	60	30
21	120	1.87
22	120	7.5
23	120	7.5
24	60	30
25	0	0
26	0	0
27	0	0
28	0	0
29	0	0
30	1.87	0.9
31	1.87	0.5
32	1.87	3.75
33	1.87	3.75
34	1.87	3.75
35	3.75	0.9
36	3.75	15
37	3.75	15
38	3.75	3.75
39	3.75	3.75
40	3.75	3.75
41	3.75	3.75
42	3.75	3.75
43	3.75	1.87
44	3.75	1.87

45	7.5	15
46	7.5	7.5
47	7.5	7.5
48	7.5	7.5
49	7.5	7.5
50	15	30
51	15	15
52	15	30
53	15	30
54	15	15
55	30	30
56	30	30
57	30	30
58	30	30
59	30	30
60	30	30
61	30	30
62	60	15
63	60	120
64	60	120
65	60	120
66	60	120
67	120	30
68	120	30
69	120	120
70	120	120
71	120	120
