# Supplementary material for: Assessing anti-rabies vaccine response in humans: A rapid and high-throughput adaptable, pseudovirus-based neutralization assay as an alternative to rapid fluorescent focus inhibition test (RFFIT)
Source: PLoS Negl Trop Dis. 2025 Apr 10;19(4):e0013010. doi: 10.1371/journal.pntd.0013010 (PMC12013880; doi:10.1371/journal.pntd.0013010)
Supplement: S1 Data — (DOCX) [file pntd.0013010.s002.docx]

**Assessing anti-rabies vaccine response in humans: A rapid and high-throughput-adaptable, pseudovirus-based neutralization assay as an alternative to Rapid Fluorescence Focus Inhibition test (RFFIT)**

Santhik S Lupitha^1^, Geetu Rose Varghese^1^, Lekshmi J Das^1^, Priya Prabhakaran^2^, Ashwini M. Ananda^3^, Reeta S Mani^3^*, Easwaran Sreekumar^1,4^*

**Supporting Information-Raw data used for preparation of Graphs**

**Fig.4: Generation and evaluation of R-PV-SEAP**

**Figure 4C.** SEAP activity in the culture supernatants HEK293T target cells upon infection with increasing concentrations of R-PV-SEAP pseudovirions 24h post-infection.

Data

| Pseudovirion titre (TCTD_50_) | Absorbance at 405nm | |
| --- | --- | --- |
| 25. | 0.92 | 0.86 |
| 50. | 1.56 | 1.46 |
| 100. | 2.43 | 3.51 |
| 200. | 3.01 | 3.96 |
| 500. | 3.69 | 4.08 |
| 1000. | 3.87 | 4.10 |
| 2000. | 4.05 | 4.12 |

**Figure 4D .** Neutralization efficiency of anti-Rabies virus G monoclonal antibody (17C7) and a negative control antibody (4G2) at 1:64 dilution against varying concentrations of R-PV-SEAP pseudovirions.

Data

| 25TCID_50_ | | 50TCID_50_ | | 100TCID_50_ | | 200TCID_50_ | |
| --- | --- | --- | --- | --- | --- | --- | --- |
| 0.13 | 0.13 | 0.13 | 0.13 | 0.13 | 0.13 | 0.14 | 0.15 |
| 0.92 | 0.86 | 1.56 | 1.46 | 2.43 | 3.51 | 3.01 | 3.96 |

| 500TCID_50_ | | 1000TCID_50_ | | 2000TCID_50_ | |
| --- | --- | --- | --- | --- | --- |
| 0.15 | 0.17 | 0.20 | 0.24 | 0.24 | 0.33 |
| 3.69 | 4.08 | 3.87 | 4.10 | 4.05 | 4.12 |

**Fig.5A. Neutralization efficiency of R-PV-SEAP by reference antibodies and human serum samples with varying RFFIT titre**

Data

| Antibody dilutions | Rabies G antibody-17C7 (%) | | Control Antibody (%) | |
| --- | --- | --- | --- | --- |
| 64. | 98.00 | 98.50 | 5.00 | 11.00 |
| 128. | 97.00 | 97.40 | 5.00 | 0.00 |
| 256. | 98.00 | 98.50 | 6.00 | 14.00 |
| 512. | 98.50 | 99.00 | 0.00 | 4.00 |
| 1024. | 98.50 | 99.00 | 12.00 | 5.00 |
| 2048. | 98.00 | 98.00 | 1.00 | 8.00 |
| 4096. | 97.40 | 98.00 | 0.00 | 0.00 |
| 8192. | 94.00 | 95.00 | 0.00 | 4.00 |
| 16384. | 84.00 | 86.00 | 4.00 | 12.00 |
| 32768. | 63.00 | 67.00 | 0.00 | 4.00 |
| 65536. | 5.00 | 16.00 | 4.00 | 0.00 |

**Figure 5C.** Neutralization efficiency of human serum samples from rabies vaccinated individuals with varying levels of Rabies Fluorescence Focus Inhibition Test (RFFIT) positivity against 100 TCID_50_ of R-PV-SEAP pseudovirions

Data

| Serum dilutions | RFFIT<0.11 | | RFFIT<0.11 | | RFFIT<0.11 | | RFFIT-1.8 | | RFFIT-1.8 | |
| --- | --- | --- | --- | --- | --- | --- | --- | --- | --- | --- |
| 64. | 0. | 0. | 21. | 20. | 20. | 16. | 87. | 87. | 82. | 81. |
| 128. | 6. | 6. | 14. | 13. | 24. | 19. | 71. | 70. | 71. | 71. |
| 256. | 6. | 2. | 17. | 18. | 22. | 18. | 48. | 46. | 57. | 57. |
| 512. | 9. | 6. | 22. | 23. | 24. | 20. | 36. | 37. | 45. | 45. |
| 1024. | 9. | 6. | 25. | 26. | 28. | 17. | 36. | 37. | 40. | 41. |
| 2048. | 7. | 5. | 15. | 13. | 6. | 2. | 31. | 32. | 26. | 28. |
| 4096. | 7. | 3. | 17. | 12. | 5. | 3. | 20. | 16. | 22. | 24. |
| 8192. | 4. | 5. | 9. | 12. | 7. | 7. | 10. | 13. | 15. | 19. |

| RFFIT-3.75 | | RFFIT-3.75 | | RFFIT-7.5 | | RFFIT-7.5 | | RFFIT-7.5 | |
| --- | --- | --- | --- | --- | --- | --- | --- | --- | --- |
| 100. | 100. | 100. | 100. | 100. | 100. | 100. | 99. | 100. | 100. |
| 100. | 100. | 100. | 100. | 100. | 100. | 100. | 99. | 100. | 100. |
| 100. | 100. | 100. | 100. | 100. | 100. | 100. | 99. | 100. | 100. |
| 100. | 100. | 98. | 98. | 98. | 99. | 100. | 99. | 100. | 100. |
| 99. | 99. | 91. | 91. | 93. | 95. | 98. | 97. | 100. | 100. |
| 85. | 92. | 67. | 65. | 62. | 60. | 90. | 89. | 100. | 100. |
| 67. | 70. | 21. | 17. | 27. | 23. | 75. | 74. | 92. | 92. |
| 46. | 52. | 23. | 21. | 24. | 21. | 73. | 72. | 78. | 78. |

**Figure 6A & B.** Concordance of Pseudovirus neutralization titres (PVNT) with RFFIT titers

Data

| RFFIT >0.11 | RFFIT-1.8 | RFFIT-3.75 | RFFIT-7.5 | RFFIT-15 | RFFIT-30 | RFFIT->30 |
| --- | --- | --- | --- | --- | --- | --- |
| 0.00 | 87.00 | 100.00 | 100.00 | 98.00 | 100.00 | 98.00 |
| 21.00 | 80.00 | 100.00 | 100.00 | 100.00 | 100.00 | 99.00 |
| 17.00 | 48.00 | 100.00 | 99.00 | 100.00 | 100.00 | 99.00 |
| 12.00 | 59.00 | 100.00 | 97.00 | 100.00 | 99.00 | 98.00 |
| 20.00 | 100.00 | 98.00 | 99.00 | 100.00 | 99.00 | 98.00 |
| 0.00 | 76.00 | 100.00 | 100.00 | 100.00 | 99.00 | 98.00 |
| 14.00 | 100.00 | 100.00 | 100.00 | 100.00 | 100.00 | 99.00 |
| 11.00 | 100.00 | 100.00 | 100.00 | 100.00 |  | 96.00 |
| 27.00 | 100.00 | 100.00 | 100.00 | 100.00 |  | 98.00 |
| 0.00 |  | 100.00 | 100.00 | 100.00 |  | 99.00 |
|  |  |  |  |  |  | 98.00 |
|  |  |  |  |  |  | 98.00 |
|  |  |  |  |  |  | 100.00 |
|  |  |  |  |  |  | 100.00 |
|  |  |  |  |  |  | 98.00 |

**Figure 6C.**  Correlation of pseudovirus neutralization titres (PVNT) and RFFIT titres of 71 human serum samples.

**(a) Data**

| PVNA (IU/ml) | RFFIT (IU/ml) | PVNA (IU/ml) | RFFIT (IU/ml) | PVNA (IU/ml) | RFFIT (IU/ml) | PVNA (IU/ml) | RFFIT (IU/ml) |
| --- | --- | --- | --- | --- | --- | --- | --- |
| 0.00 | 0.00 | 120.00 | 120.00 | 3.75 | 3.75 | 30.00 | 30.00 |
| 0.00 | 0.00 | 7.50 | 120.00 | 3.75 | 3.75 | 120.00 | 60.00 |
| 0.00 | 0.00 | 120.00 | 120.00 | 1.80 | 3.75 | 120.00 | 60.00 |
| 0.00 | 0.00 | 30.00 | 60.00 | 1.80 | 3.75 | 120.00 | 60.00 |
| 0.00 | 0.00 | 0.00 | 0.00 | 15.00 | 7.50 | 120.00 | 60.00 |
| 1.00 | 1.87 | 0.00 | 0.00 | 7.50 | 7.50 | 120.00 | 60.00 |
| 1.87 | 1.87 | 0.00 | 0.00 | 7.50 | 7.50 | 120.00 | 120.00 |
| 0.50 | 1.87 | 0.00 | 0.00 | 7.50 | 7.50 | 120.00 | 120.00 |
| 0.50 | 1.87 | 0.00 | 0.00 | 7.50 | 7.50 | 120.00 | 120.00 |
| 0.90 | 7.50 | 0.90 | 1.87 | 30.00 | 15.00 | 30.00 | 120.00 |
| 7.50 | 7.50 | 0.50 | 1.87 | 15.00 | 15.00 | 30.00 | 120.00 |
| 7.50 | 7.50 | 3.75 | 1.87 | 30.00 | 15.00 |  |  |
| 7.50 | 7.50 | 3.75 | 1.87 | 30.00 | 15.00 |  |  |
| 7.50 | 7.50 | 3.75 | 1.87 | 15.00 | 15.00 |  |  |
| 15.00 | 15.00 | 1.00 | 3.75 | 30.00 | 30.00 |  |  |
| 7.50 | 15.00 | 15.00 | 3.75 | 30.00 | 30.00 |  |  |
| 7.50 | 15.00 | 15.00 | 3.75 | 30.00 | 30.00 |  |  |
| 7.50 | 15.00 | 3.75 | 3.75 | 30.00 | 30.00 |  |  |
| 15.00 | 15.00 | 3.75 | 3.75 | 30.00 | 30.00 |  |  |
| 60.00 | 60.00 | 3.75 | 3.75 | 30.00 | 30.00 |  |  |

**(b) Analysis**

| Pearson r |  |
| --- | --- |
| r | 0.9249 |
| 95% confidence interval | 0.8803 to 0.9533 |
| R square | 0.8555 |
|  |  |
| P value |  |
| P (two-tailed) | < 0.0001 |
| P value summary | **** |
| Significant? (alpha = 0.05) | Yes |

**Fig.6 D. Receiver operator characteristics (ROC) of the PVNT assay**

| 1-Specificity | | Sensitivity |
| --- | --- | --- |
| 0. |  | 0.89 |
| 0. |  | 0.89 |
| 0. |  | 0.91 |
| 0. |  | 0.94 |
| 0. |  | 0.95 |
| 0. |  | 0.95 |
| 0. |  | 0.97 |
| 0. |  | 0.97 |
| 0. |  | 0.98 |
