# Supplementary material for: Assessing anti-rabies vaccine response in humans: A rapid and high-throughput adaptable, pseudovirus-based neutralization assay as an alternative to rapid fluorescent focus inhibition test (RFFIT)
Source: PLoS Negl Trop Dis. 2025 Apr 10;19(4):e0013010. doi: 10.1371/journal.pntd.0013010 (PMC12013880; doi:10.1371/journal.pntd.0013010)
Supplement: Attachment — Submitted filename: 1 Response to Reviewers-Final.docx [file pntd.0013010.s004.docx]

**Response to Reviewers' comments:**

We are extremely thankful to the reviewers for their critical reading of the manuscript and the insightful comments. Our responses to each of the comments are given below.

**Methods**

**Reviewer #1:**

The methods were clearly stated to accomplish the stated goal of development of a valid rabies pseudotype neutralization assay. As weel the design was completed, including both detection in cells and supernatant. There are minor points/details and would allow improved description of the experiments:
Under Titration of R-PV-SEAp pseudovirus stocks, what serial dilutions of the stocks were used?

Response:	We used a two-fold serial dilution in 2% DMEM for the titration of the	pseudovirion stock. This has been indicated in the revision.

 In the ethics statement, was SRIG was used in the RFFIT test for the human serum samples tested?

Response:	Yes, SRIG was used in the RFFIT test for the human serum samples tested.

Which WHO reference serum was used in the PVNT assay, was it the same WHO reference serum used to qualify the 17C7 monoclonal?

Response:	WHO Reference serum (30IU/mL) obtained from National institute of Biological	standards and Control (NIBSC), UK was used in the RFFIT assays. 17C7 monoclonal	was titrated against the same WHO reference serum.

In the paragraph where the PVNT is first described, it mentions that the samples were diluted in 2% FBS containing DMEM but there is no mention of what diliution was used. In 2 paragraphs later a 2-fold serial dilution was performed, referencing Smith, 1973 (the basis of the RFFIT method in the WHO manual) states a 5-fold serial dilution is used. Suggest, referencing the modified method (Ashwath) only.

Response:	We used a two-fold serial dilution of the antibody in all the PVNT assays as per the	modified protocols of the RFFIT. So, as suggested by the reviewer, we modified the	cited references and included only the relevant one.

What was the cell count used for the HEK293T cells?

Response:	We used HEK293T cell at a concentration 20000 cells/well for seeding the 96 well	plate. After 24 hrs of incubation, once the monolayers were formed, the medium was	removed and the monolayer was infected with the pseudovirus. The details have been	included in the revised manuscript.

**Reviewer #2**: The article “Assessing anti-rabies vaccine response in humans: A rapid and high-throughput— A rapid and high-throughput-adaptable neutralization assay as an alternative to Rabies Fluorescence Focus Inhibition test (RFFIT)” describes a more practical alternative to RFFIT that avoids using live rabies virus, a fluorescence microscope, and uses SEAP expressed by the pseudovirus as a reporter.
Dear authors, congratulations on the submitted manuscript. It is well presented, and the work represents a very interesting alternative system to measure rabies-neutralizing antibodies.

The objectives and study design it is appropriate.

When reading, I was expecting also a comparison with the R-PV-GFP virus.

Response:	We are very glad for the encouraging comments and thank you so much. In our study,	we primarily used the R-PV-GFP to confirm the pseudovirion production and the	optimization of target cells for the infection. This also helped us to visualize the	pseudovirions by immunostaining of the viral particles produced giving confidence on	this new approach.

However, once we made sure that the pseudovirion generation is working, we wanted	to address some of the limitations of the current RFFIT assays, such as the need for a	fluorescence-based system for visualization, making it difficult in resource-limited	settings; as well as non-adaptability for high-throughput applications. So, we focused	more on the SEAP-based assay generation and validation. We are hopeful that the	SEAP-based assay will get more acceptability for a wider use.

At a technical level, both the GFP-based and SEAP-based system should work equally	well with respect to neutralization specificity as both uses the same Rabies-G protein	as the surface glycoprotein; and the difference is only in the reporter in the recombinant	genome of the VSV virus backbone. However, since GFP detection uses microscopic	visualization, it will be able to detect even single cell infection, making it more	sensitive as RFFIT. We have discussed the compromised sensitivity of the SEAP-based	system in the manuscript.

**Results**
**Reviewer #1:** The result section described the outcome of the generation of the pseudotypes and the ability of the pseudotypes to measure neutralization by previously determined positive RVNA samples. The figures are effective in displaying the results. Under the section Susceptibility of R-PV-SEAP to neutralization by reference.... the cut-offs used for the assay are not discussed, It would be helpful also if descriptions of high, low, borderline and negative RFFIT ranges were given.

Response:	As suggested by the reviewer, we have included the cut-off values used. As this was	done in a small subset of 10-samples and was the initial evaluation, we used here a	100% neutralization cut-off. However, as described in the section that follows, when	we increased the sample size, we could lower this cut-off value to even 50% without	affecting the positive predictive value. Also, the RFFIT values based on which we had	classified the samples to high, low, border-line and negative have been indicated	in the revised section.

At the bottom of page 11 the sentence: The assay showed con characteristics such as..., what is meant by con?

Response:	This is a typographical error and our apologies for the confusion. The word intended	was 'consistent'. We have corrected the same in the revised manuscript.

**Reviewer #2**: When reading, I was expecting also a comparison with the R-PV-GFP virus.

Response:	As explained above, our focus was to develop an assay with an easy read out and high	throughput adaptability. So, in this manuscript, we have not ventured into a detailed	comparative analysis of R-PV-GFP and R-PV-SEAP. However, we will be happy to	undertake such a study in due course.

Please find some small suggestions below, based on the pdf version received:

Page 1) Title: Please check that two words are together, at least in the loaded file.

Response:	We think it is a formatting error formed while generating the pdf file during	submission. We will confirm that it is not present in the final version

Page 7) In “Titration of R-PV-SEAP," when mentioning “serial dilution," which dilution are you referring to? Twofold or tenfold dilution?

Response:	We did a two-fold dilution. This has been mentioned in the revised version.

As far as I understand, the experiments are done in preformed cell monolayers; is correct?

Response:	Yes. We did the infection experiments in the monolayer cultures. The cells are seeded	on the previous day, and after 24h, infection is done. The details have been	included in the revised version.

Page 10) Please check whether R-PV-SEAP should replace RPV-SEV.

Response:	It was a typographical error. This has been corrected in the modified version.

Page 13)

When you mention that the titer reached with the R-PV-GFP is produced in high titer, are the same numbers as the R-PV-SEAP? Could you add this number to the discussion?

Response:	We could produce the R-PV-GFP to a titre of 1.2- 2.0 × 10^5^ FFU /ml. This number has	been included in the discussion section.

At which scale is it enough to prepare a viral stock for an X quantity of rabies-neutralizing antibody determinations? Just a comment, if possible.

Response:	From a 60-80% confluent culture of Rabies G plasmid-stably transfected HEK293	producer cells in T-25 flasks, we can prepare 4ml of R-PV-SEAP pseudovirion	supernatant containing about 50000-63000 TCID_50_/mL after infection with ΔG-GFP	(G*ΔG-	SEAP) rVSV at approx.3-5 MOI. 1 ml of this will be sufficient to do a two-	fold dilution (from 1: 64) assay with eight-points for around 50-60 serum samples at	100 TCID_50_ virus per assay. So, a total of 200-250 samples can be tested by the 4 ml	pseudovirus stock virus produced.

Page 18)

In Figure 1, or the manuscript, the MOI (or infection dilution used) is not mentioned.

Response:	2×10^4^ FFU R-PV-GFP pseudovirion particles were used to infect the target cells	grown in 96-well plates at a MOI of 1. The details are included in the revised	manuscript.

Figure 1.A) Consider if it could be improved for those unfamiliar with the system.

Response:	We have added a new summary scheme figure (Fig.7) indicating the experiments

including more details so that the system becomes clearer to those who are unfamiliar.

Page 21)

In Figure 3, what is the titer of the virus mixed with the serum?

Response:	Two-fold dilutions of the serum in 100μl of 2% FBS containing DMEM were pre-	incubated with an equal volume of the medium containing 2×10^4^ R-PV-GFP	pseudovirion particles for 1hr at 37ºC; and were used to infect monolayers of	HEK293T target cells cultured in 96-well plates. The figure legend has been modified	accordingly.

**Conclusions**

**Reviewer #1:** The discussion method is written very well, and effectively expresses the reason for the study and why use of a rVSV-based system for obtaining high titre is preferred. The practical advantages were well covered. The importance of a rabies antibody assay having high specificity in spite of low sensitivity was adequately made. Perhaps the "equal or better" statement was overstated.

Response:	Thank you for the positive observations. We have modified the statement replacing the	wording ‘equal or better’ with the wording ‘comparable'.

Some of the downsides to the traditional RFFIT, while experience in some hands, does not apply to all laboratories that can report values within 24 to 48 hours, and not experience statin bleaching with new efficiency since the 1970s. Indeed, SNs can and are be read by instrumentation (Wu et al. JVirolMeth 2023 for example) --a more evened argument would be more factual. However, the main advantage is elimination of need for high containment was well covered in light of the zero by 30 effort.

Response:	We have modified this section and tried to present a more evened argument in the	revised	manuscript.

**Reviewer #2:** Page 14) Besides the limitations you mentioned about the current state of this work, does R-PV-GFP work similarly to the R-PV-SEAP? Immunostaining should not be necessary because it expresses the GFP reporter well.

Response:	We agree with the reviewer that the GFP-based system will be able to avoid the	immunostaining step; and definitively will be an improvement to the existing assay.	Also, R-PV-GFP will be more sensitive compared to R-PV-SEAP because of the	microscopic detection. R-PV-GFP will work equal compared to RFFIT, but both lacks	high-throughput capability and needs fluorescence-based visualization systems. So, we	presume that the SEAP-based assay will have its own advantages.

If you have information or data (using R-PV-GFP) similar to the one generated with the R-PV-SEAP, it could be of interest to those who are already doing RFFIT and have access to a fluorescence microscope.

Response:	At present, we have not generated more data with the R-PV-GFP-based system	especially in the assays with human samples. However, as the reviewer has indicated,	it will be a useful study and we will plan experiments to do this in due course to have	a comparative analysis of both the systems.

**Editorial and Data Presentation Modifications?**

**Reviewer #1:** Minor comments:

In the Abstract, correct the name of RFFIT, it is Rapid Fluorescent Focus Inhibition test (see WHO Manual).

Response:	Thank you for pointing this out. We have corrected the same in both the abstract as	well as the title to conform with the usage in WHO manual. As we had seen some	manuscripts using the term 'Rabies' instead of the word 'Rapid', we were under the	impression that both terminologies can be used.

Clarify the sentence in the Abstract: In samples with border-line RFFIT values (what are borderline?), concordance was low, nevertheless, the results were tending to be negative (waht results, borderline RFFIT or PVNT results?).

Response:	A RFFIT titre 0.5 -1.8 IU/ml was considered as borderline RFFIT positivity; and in	these samples, the PVNT tests were negative. We have made modifications in the	abstract section to reflect this.

Using the term protection in terms of rabies antibody level should be defined. There is no known/proven level of rabies antibody, the 0.5 IU/mL was assigned by review of response to vaccination and represents a robust level.

Response:	We agree with the reviewer. We have removed the term 'protection' and modified the	sentence accordingly.

In the Introductions the last sentence reads oddly. Perhaps what is meant is RFFIT-validated rabies antibody levels in human serum samples.....

Response:	We have modified this sentence to improve clarity.

**Reviewer #2:** (No Response)

**Summary and General Comments**

**Reviewer #1:** Well-written, worth study to be published. Expected to be useful to the zero by 30 effort and generation of useful data for future studies and policies.

Response:	We are happy for the positive comments and thank you so much

**Reviewer #2:** (No Response)

**-------------------------**
